# Canonical Insertion-Deletion Markers for Rapid DNA Typing of *Francisella tularensis*

**DOI:** 10.3201/eid1311.070603

**Published:** 2007-11

**Authors:** Pär Larsson, Kerstin Svensson, Linda Karlsson, Dimitri Guala, Malin Granberg, Mats Forsman, Anders Johansson

**Affiliations:** * Swedish Defence Research Agency, Umeå, Sweden; † Umeå University, Umeå, Sweden

**Keywords:** Bacteriology, DNA fingerprinting, VNTR loci, indel loci, Francisella tularensis, molecular epidemiology, genetic speciation, molecular phylogeny, classification, research

## Abstract

To develop effective and accurate typing of strains of *Francisella tularensis,* a potent human pathogen and a putative bioterrorist agent, we combined analysis of insertion-deletion (indel) markers with multiple-locus variable-number tandem repeat analysis (MLVA). From 5 representative *F. tularensis* genome sequences, 38 indel markers with canonical properties, i.e., capable of sorting strains into major genetic groups, were selected. To avoid markers with a propensity for homoplasy, we used only those indels with 2 allelic variants and devoid of substantial sequence repeats. MLVA included sequences with much diversity in copy number of tandem repeats. The combined procedure allowed subspecies division, delineation of clades A.I and A.II of subspecies *tularensis*, differentiation of Japanese strains from other strains of subspecies *holarctica,* and high-resolution strain typing. The procedure uses limited amounts of killed bacterial preparations and, because only 1 single analytic method is needed, is time- and cost-effective.

## Abstract 2

By combining analysis of indel markers with multiple-locus variable-number tandem repeat analysis, individual strains were identified.

*Francisella tularensis* is a highly infectious, facultative intracellular pathogen and the causative agent of the zoonotic disease tularemia. Based on virulence tests and biochemical assays, *F. tularensis* is divided into 4 subspecies, a division that has recently been corroborated by genetic typing (*1*,*2*). Each subspecies shows a discrete natural geographic distribution and also varying degrees of virulence (*3*). Human disease caused by *F. tularensis* subsp. *tularensis* may be fulminate or even lethal, whereas disease caused by other subspecies is less severe, although often incapacitating and protracted (*4*). In addition, recent molecular and epidemiologic analyses of natural isolates of *F. tularensis* subsp. *tularensis* suggest a population split of the subspecies into 2 major groups of isolates, which differ in virulence and geographic distribution (*5*–*7*).

Robust and rapid typing schemes for *F. tularensis* are needed, not only because of their use in clinical and public health work but also because of a rising concern associated with risks for bioterrorism (*4*,*8*). Because of its virulence, *F. tularensis* is included among the top 6 “category A” potential bioterrorism agents believed to have the greatest potential for adverse public health effect with mass casualties. If deliberate release of the organism is suspected, the need to understand the pathogenic potency of an isolate and also its putative origin will be urgent.

In standard medical practice, subspecies determination of *F. tularensis* typically involves biochemical fermentations. Such analyses are labor-intensive, hampered by the fastidious growth characteristics of the organism on artificial media, and associated with a substantial risk for laboratory-acquired infections (*2*,*9*).

Several DNA-based methods have been found useful for typing of *F. tularensis* at the subspecies level (*1*,*10*–*13*). Among these, pulsed-field gel electrophoresis (PFGE) is more widely adopted and was recently proposed for diagnostic and epidemiologic work on *F. tularensis* by PulseNet laboratories throughout the United States (*7*). PFGE typing is, however, far from ideal for the purpose. It involves making concentration-adjusted suspensions of live bacteria, which has the potential for creating infectious aerosols, is time-consuming, produces complex banding pattern data, and has a restrictive discriminatory capacity when applied to *F. tularensis* (*7*,*14*–*17*).

High-resolution typing of *F. tularensis* is currently attainable only by the use of multilocus variable-number tandem repeat analysis (MLVA). The method capitalizes on differences among strains in copy numbers of sequence repeats at multiple genomic loci. MLVA has been successfully applied in epidemiologic studies on tularemia (*5*,*6*,*18*,*19*). Killed bacterial preparations can be used in the assay and, in contrast to PFGE, MLVA produces discrete-character numeric data, which are well suited for easy transfer among laboratories. For discrimination of strains of *F. tularensis*, MLVA is the obvious choice.

A limitation inherent in MLVA is the risk for erroneous estimates of relationships among strains at larger genetic distances. The high rates at which MLVA markers mutate (*20*,*21*), and possible functional constraints on these sequences, may cause homoplasy effects, i.e., share of mutational changes for reasons other than common ancestry (*22*,*23*), implicating a risk for spurious strain affiliation. In work on *Bacillus anthracis*, the issue was addressed by analysis of single-nucleotide polymorphisms (SNPs), which exhibited canonical properties for resolving major genetic lineages (*24*). In a hierarchical typing approach, which conformed with concepts of traditional bacterial taxonomy, a 2-step procedure was suggested, including assay of canonical SNPs for resolution of major genetic clades and MLVA for high-resolution typing (*24*). A limitation of the procedure is that it involves 2 assays, thus increasing time and cost.

When aiming to construct an improved typing strategy for *F. tularensis*, we focused on insertion-deletion (indel) markers. By definition, indels are caused by insertion or deletion of >1 base pairs of a DNA molecule. Among indels, the evolutionary rates diverge widely. When used as a complement to MLVA, more slowly evolving indels, i.e., loci displaying a relatively low degree of variability, would be preferable. A practical reason to use canonical indel markers was that fragment analysis can by used for simultaneous assay of both indel and MLVA markers, thereby minimizing time and cost.

We identified indel markers with canonical properties in *F. tularensis* and used them to resolve major genetic lineages of the species. We also developed a strategy that combines indel analysis with MLVA for rapid and accurate discrimination of isolates of the species.

## Material and Methods

### Genome Sequences, Strains, and DNA Preparations

We used genome sequences for the 5 strains, U112 (aka FSC040, ATCC 15482), FSC147 (GIEM 543), SCHU S4 (FSC237), OSU18, and LVS (FSC155) (Appendix Table 1), for in silico work, and in total, 23 isolates ( Appendix Table 2, Appendix Table 3) were selected for the experimental work. These were chosen to represent each of the 4 currently recognized *F. tularensis* subspecies and were selected from the *Francisella* Strain Collection (FSC) maintained at the Swedish Defence Research Agency, Umeå, Sweden. Bacteria were grown on modified Thayer-Martin agar (*25*), suspended in phosphate-buffered saline, and immediately heat killed. DNA was prepared by using silica and guanidine isothiocyanate buffer (*26*). Extended information on strains and, when appropriate, GenBank accession numbers, are available in Appendix Tables 1–3.

### Identification and Selection of Indel Markers

Multiple alignment of genomic sequences for *F. tularensis* strains U112, FSC147, SCHU S4, OSU18, and LVS was performed by using Mauve 2.0 β multiple alignment software (*27*) and the progressive alignment option. The output file produced by Mauve was parsed by using a custom Perl script to retrieve multiple aligned sequences for indel loci that fulfilled the following criteria: 1) the loci should exist in all compared strains, 2) only 2 allelic variants should exist, 3) at least 25 bp of sequences lacking other indels should flank identified loci, 4) indels should be 5- to 200-bp long, and 5) direct repeated sequences of substantial length should not be present at indel loci because such sequences may increase the risk for homoplastic mutation.

### Primer Design and PCRs

Oligonucleotide primers for PCR amplification were designed by using the Primer3 tool (*28*) and a Perl script to supply aligned sequences and required coordinate information. To reduce experimental cost, the forward primer of each primer pair was synthesized with an additional 19-bp M13 tail added to the 5′ end of the primer (Table). This enabled the use of fluorescently labeled M13 PCR primers to simultaneously amplify marker loci and label the PCR amplicons. The M13 primers were labeled terminally with D2-PA, D3-PA, or D4-PA dyes at the 5′ end (Proligo Primers and Probes, Hamburg, Germany).

**Table:** Insertion-deletion loci, genomic locations, and primers

Ftind locus*	Positions†	Pattern	Forward primer sequence (5′→3′)‡	Reverse primer sequence (5′→3′)
1	1152573–1152844	12222	TCTCGTGACAGAGCTTTACAA	GGGAGAATTGATTATGGCTTAC
2	895732–896067	12222	AGCAGCGTATCGAAGAGATAG	TAAATCTAGTTGGCTGAGTAATAAAGTC
3	769704–770059	12222	CAAACCTAATTGCTCCAGAAC	GCAGCATATCTTTGGTCATCTAT
4	520340–520556	12222	TTTGAAAAGCTAGAAAAAGATGC	ACCAAGAATATTAAAAGCCAAATC
5	1628363–1628558	12222	AACTAAGTTGTTTTAGTGGGTTCC	CAATTTTATACCCCAGTTAATATTTGA
6	562346–562675	12222	CAACAATCTCACCATTACCTAAAA	GCTAGGCAAGCCATTATATTTATC
7	688418–688771	12222	CCAAAATATACCAAAATATCCTATCA	ATTTATGCAATATCACAAGTTCCA
8	198167–198521	12222	GTGACCTAATCAAAGAGCAACTAA	ATCTGCATACTTGAGTAAATGCTT
9	1830520–1830768	11211	CTCAAGAAATTAAAGGGATGAGTT	ATTTGCTCAGTACCTGCTAATGTA
10	1113820–1114081	11211	CATTCCTAGTRATAGCTCCTGCT	ATTAAGCTTCAACACTATCATCATCT
11	1238526–1238784	11211	TACTTTTAATGCTTCAGCGACA	AATCACCAATAACCCAGACAAC
12	725006–725258	11211	GCCTATGCTGGTAAAGTTGG	TCACCAATAGCTTCCATAACAC
13	1490938–1491179	12211	AACTCCTGGTTTCCCACAC	GCTACAAAACTCACTATGTTCAGAC
14	625186–625399	12211	GACTGAACAACAACTGGATTATCAC	TGTAGTCCATTAGGGCAGTAATCTT
15	573074–573303	12111	GGTTTTGTTGCTAAATCTGC	ACGCTGATCATCAATCATTC
16	1628145–1628393	12111	TCCTTTAAAGAAACGGCATA	TCTGTACGGAACCCACTAAA
17	239966–240157	12111	CATGAAAACTTGGTTATAGCTGA	GCGCAAGATCAGCTTAGTT
18	439229–439434	12111	AGAGTTAACCCATTCAACAAGA	GGCAAGGTTTCTGGATAGAC
19	408363–408515	12111	TTTGATAGCTCAAATGCAAGA	AGCTAGCTTGCCTCTTTTCT
20	602863–603177	11122	AAATCATTTAACAATTGGTATCTTT	TAGCTCTGAGTTAGAAAAACTCG
21	271531–271863	11122	TCTTCTTGTATAAGATGCGCTAAA	GGTTAAGTTAGGGCAATGTAAGAT
22	5648–5976	11122	TGACAAAGAAGACTAAGCACAAAT	GGTTTGATAAATGCAAACTATATGAT
23	1062332–1062553	11122	TCAACCGGCTTTATGAGAGTA	TATTACGAGACCGAAAATACGATA
24	1641399–1641720	11122	AATTCAAAAAGCGATAAGTAACCT	GCCAGCAACATACTCTTTTGT
25	267938–268267	11122	AAATTAAAGCAAGGACAGGTTTAT	TCCATAGTTATTTCAACTTGGTTT
26	1828819–1829145	11122	AGCTGCTAAATCTAAACTCTTTGC	GCTCCCTCAACTAGATCTATCATC
27	960872–961191	11122	AATCGCATACATTTCTGCTGTA	GCTTTTCCAAATGAGGATATTAAA
28	1136267–1136582	11122	AAAAGTAGCTGCAGAAGTATACCC	TTCTCAAAATGTAAACATGCTTCT
29	1190422–1190738	11122	CTTGAGCTTACGCCCTTTTAT	ATGTCCGCAATATTGTCCTAAC
30	871284–871614	11112	CTGCATTTTCAACATTACTCAGAT	ATTCATAAAGATCATCCATTCCTC
31	518787–519092	11112	AGCTGTAGTGATATAAAGAAAAGTTACAT	CTATTTCGTAGCGAGTAAGAATTT
32	1709427–1709741	11112	TTATGCAAATAACTATCCAAGTGTT	TTACCATTAGCTTCAAAAGTCTGT
33	511958–512251	11112	TACAAGCGTACCATCTAAGTCA	CATATTGGGATGTCAAGCA
34	99015–99303	11112	TTGATATAACCAACATAAACACTGC	TGAGTATAGAAATACAAAGCTACGC
35	772225–772590	11121	TGTGTAGTAACCCAGGAACTTTAT	AATTTGATGCCATATGAGAGAAT
36	282847–283070	11121	TTTGGTATGAGTATTCTGGTCCTA	GTATTTTGGTTTAGCTTACGGATT
37	1486225–1486603	11121	AATATTTGCAACCAATGATGATAC	CAGTATCTTTGATGTTAGGGACAA
38	95621–95874	11121	GCTACGACAGGTCTATCTTTCTC	CAACTTATGATTGGTGATGATGT

*Ftind, *F. tularensis* insertion-deletion marker. †Location of the DNA amplified by PCR in the chromosome of *Francisella tularensis* strain SCHU S4. ‡Sequences given for forward primers represent the target-specific parts of the primers used. For inexpensive fluorescent labeling, each forward primer was synthesized with a 19-bp extension at the 5’-end, corresponding to an M13 sequence (5′-GTAAAACGACGGCCAGT-3′).

**Figure 1:**
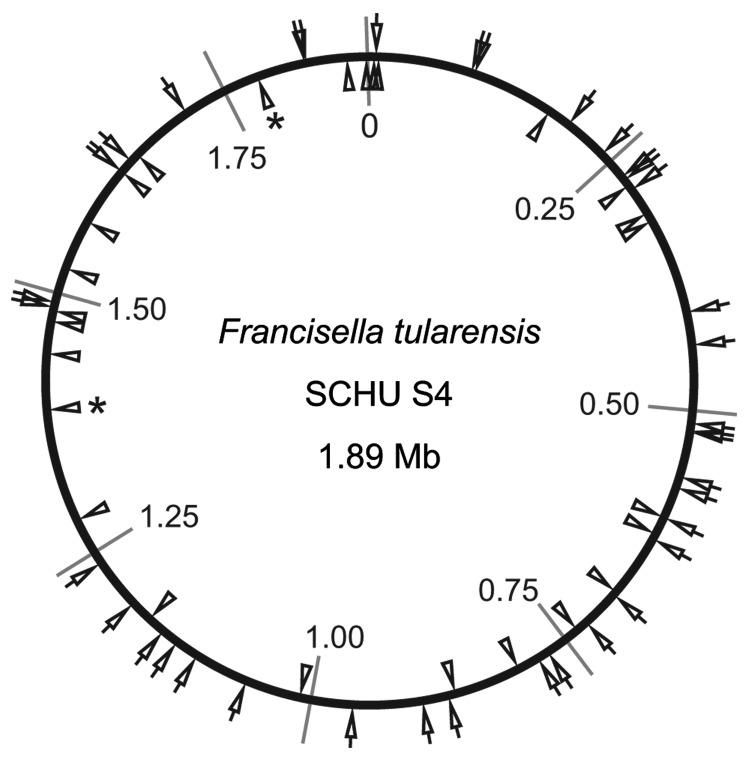
Locations of 38 insertion-deletion and 25 multilocus variable-number tandem repeat analysis (MLVA) markers on the physical genome map of *Francisella tularensis* subsp. *tularensis* strain SCHU S4. Positions are given with reference to the predicted origin of replication set at position 0. Indel and MLVA marker locations are depicted by wedges on the outside and inside of the circle, respectively. Two asterisks indicate the duplicate occurrence of the MLVA loci Ft-M14 at 2 different locations because it is part of a large sized genome duplication (*1**,**25*)*.*

PCR amplification was performed in 96-well microtiter plates. Each reaction mixture contained 0.15 mmol/L dNTP, 0.6 U DyNAzymeII polymerase (F-501L, Finnzymes, Espoo, Finland), 1 μL PCR buffer for DyNAzyme DNA polymerase (Finnzymes), 2 μL of template DNA (20 ng/μL), 0.3 pmol/L forward primer, 0.8 pmol/L reverse primer, and 0.8 pmol/L labeled M13 primer. Filtered sterile water was added to a final volume of 25 μL. The PCR reactions were performed in a MyCycler thermal cycler (BioRad, Hercules, CA, USA) with the following program: 95°C for 2 min; 15 cycles of 95°C for 30 s, 56°C for 30 s, 72°C for 45 s; 20 cycles of 95°C for 30 s, 51°C for 30 s, and 72°C for 45s; and then a 7-min final extension step at 72°C. MLVA was performed as previously described, except modified to use fluorescence-labeled forward primers (*6*). The physical distribution of 38 selected indel markers identified in this study and 25 MLVA markers throughout the genome of strain SCHU S4 (*29*) is illustrated in Figure 1.

### PCR Amplicon Separation

PCR reaction mixtures, 2 μL from each, were pooled and diluted 15-fold. One μL of diluted sample was added to 40 μL of sample loading solution, containing DNA Size Standard-600 (Beckman Coulter Inc., Fullerton CA, USA), and sealed with a drop of mineral oil. Finally, PCR amplicons were separated and detected by using a CEQ 8800 Genetic Analysis System (Beckman Coulter Inc.). Binning of indel fragment size-calls was straightforward because of highly precise size determinations ( Appendix Table 2). Maximum size divergence between size-call and genome sequence data was 3 bp among 38 selected indel markers for strains U112, FSC147, SCHU S4, or LVS.

### Statistical Analysis

Simpson’s index of diversity (1 – D) (*30*) was determined for each investigated marker as a measure of both richness and evenness, calculated as


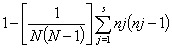


where *N* is the number of strains, *s* is the number of recorded states for a marker, and *nj* is the number of strains belonging to the *j*th marker state. Both distance-based clustering, by using hamming distance (*31*) and the neighbor-joining method, and maximum parsimony (MP) were performed with PAUP* version 4c10 (*32*). MP analyses were performed by using 50 replicates without branch swapping and 10,000 bootstrap pseudoreplicates. Nodes supported by <50% bootstrap pseudoreplicates were collapsed in depictions of the obtained consensus topologies. Indel size and distribution of repeat size frequency were analyzed by using the R statistical package (*33*).

## Results

### Identification and Selection of Indel Loci

**Figure 2:**
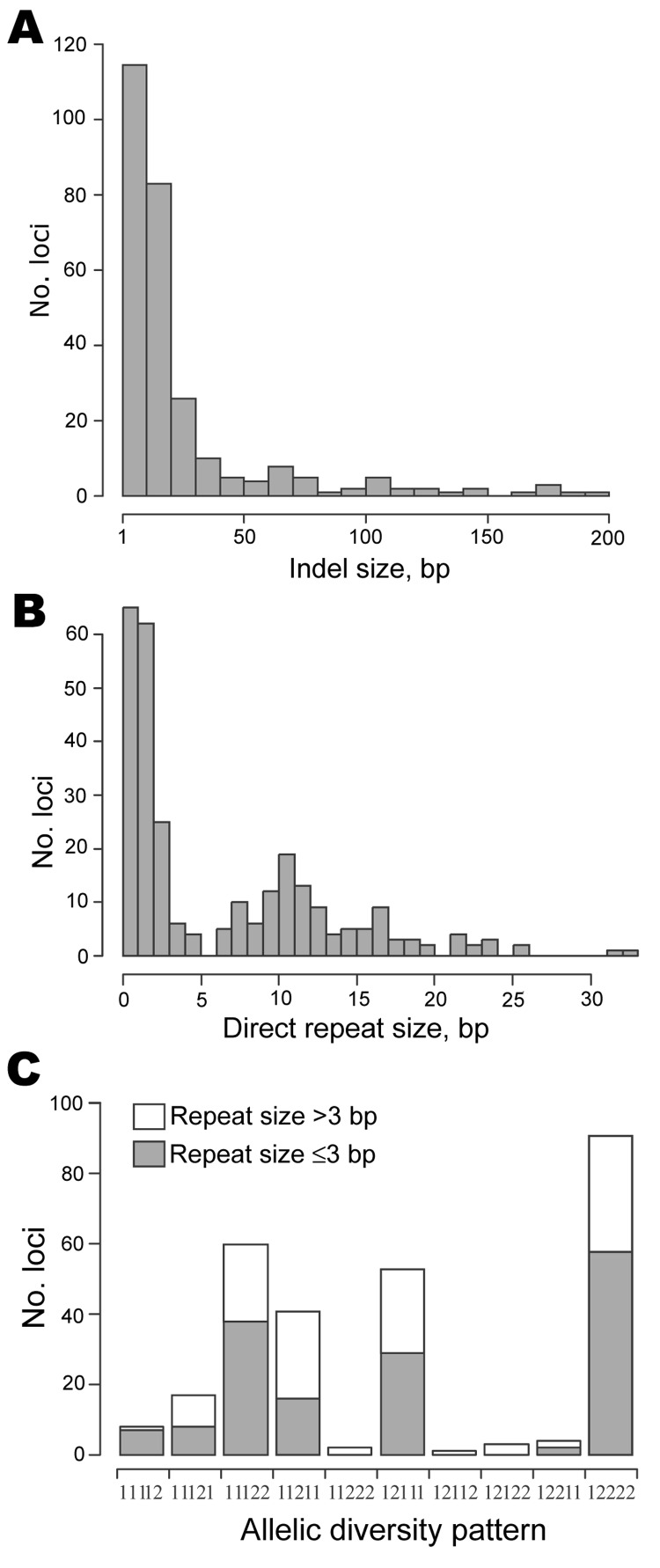
Properties of 280 insertion-deletion (indel) loci identified by analysis of 5 *Francisella tularensis* genome sequences. The diagrams show distributions of indel sizes (A), repeat sizes detected at these loci (B), and10 allelic diversity patterns (C); the number 1 or 2 represents each of the 2 allelic variants. A string of numbers includes, in order, strain U112 (subsp. *novicida*), FSC147 (subsp. *mediasiatica*), SCHU S4 (subsp. *tularensis*), OSU18 (subsp. *holarctica*), and LVS (subsp. *holarctica*). Empty and filled bars correspond to the presence or absence of repeats >3 bp long, respectively.

In the genomic sequences of each of 5 *F. tularensis* strains (Appendix Table 1), a total of 280 indel loci were identified, all exhibiting only 2 allelic variants and a size range of 5–200 bp. Small-sized indels predominated; 70% were shorter than 20 bp (Figure 2, panel A). To enable the selection of loci free from such repeat nucleotide sequences, which may have a propensity to initiate deletion or insertion mutations, indels were analyzed with regard to the size of associated repeats. Two repeat size peaks were identified, 1 at 10 bp ± 1 bp and another <3 bp (Figure 2, panel B). In 62 loci, no repeats were found. After exclusion of loci associated with repeats >3 bp in length, 158 loci were retained for typing purposes.

To facilitate selection of indel loci represented in various strains, we analyzed the diversity of the 280 allelic variants among the 5 *F. tularensis* genomes included. Among the genomes, only 10 discrete allelic diversity patterns were found, depicted in Figure 2, panel C, as allelic variant 1 or 2 in each of the genomes in order of strains U112, FSC147, SCHU S4, OSU18, and LVS (e.g., 1,2,1,1,1 denotes that a deletion was present in the genome sequence of strain FSC147, but not in any of the others). After loci associated with repeats >3 bp in length were excluded, 7 allelic patterns were retained and used as a basis for selecting indel loci for the assay (Figure 2, panel C).

By these measures, a subset of 38 loci was selected (Table;  Appendix Table 2). These loci showed maximum diversity, represented each allelic pattern among the 5 genomes, and also exhibited a physical separation on the SCHU S4 chromosome (Figure 1).

### Analysis by the Combined Procedure of 24 Strains of *F. tularensis*

Twenty-four strains, representing all 4 subspecies of *F. tularensis* and clades A.I and A.II of *F. tularensis* subsp. *tularensis*, underwent indel analysis and MLVA ( Appendix Tables 2, 3). Of these, 23 yielded indel PCR amplicons in the range of 145–399 bp, representing an allele of each of 38 loci analyzed. In the remaining strain, isolate FSC454, PCR amplification failed for 7 indel loci tested. FSC454 is an atypical *Francisella* isolate of uncertain taxonomic status recently isolated in Spain (R. Escudero, pers. comm.). FSC454 was excluded from further analyses.

Another atypical strain, ATCC 6223, yielded aberrant amplification results. This strain has lost virulence for mammals, a key characteristic of *F. tularensis*. It exhibits unusual colony morphologic features and a slow growth rate. When subjected to PCR amplification, the genome of strain ATCC 6223 yielded 2 DNA amplicons for an indel locus denoted Ftind-32. Ftind-32 and ATCC 6223 were retained for further analysis, and both alleles were considered.

**Figure 3:**
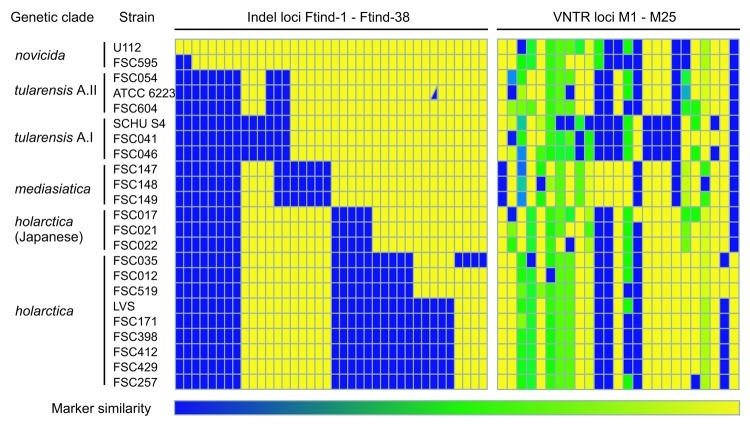
Heat map of marker states for 38 insertion-deletion (indel) and 25 multilocus variable-number tandem repeat analysis (MLVA) loci examined. Each *Francisella tularensis* strain is represented by a single row of colored boxes and each DNA loci by a single column. Relative genetic similarity is represented by the similarity of the colors on the gradient scale ranging from blue to yellow. For the binary indel markers, the state of each marker in the genome of strain *F. tularensis* subsp. *novicida* U112 represents the index and is depicted in yellow. Blue indicates the amplification of an allelic variant distinct from that of the index genome. For strain ATCC 6223, both alleles were amplified at loci Ftind-32, and the corresponding box is thus divided into a yellow and a blue part. For MLVA loci, blue represents the largest allele size for each multistate marker; yellow represents the smallest.

A graphic representation of the observed amplification patterns at indel and MLVA loci is shown in Figure 3. A difference in mutational stability was apparent between indel and MLVA loci. Indel loci showed a binary pattern that grouped *F. tularensis* in agreement with traditional taxonomy based on phenotype. In accordance with previous genetic typing by MLVA, PFGE, or sequencing of 7 housekeeping genes, the indel analysis distinguished 2 major subpopulations of type A strains (denoted A.I and A.II) and also showed Japan-derived *F. tularensis* strains to be distinct from strains of *F. tularensis* subsp. *holarctica* isolated in other parts of the Northern Hemisphere. Furthermore, indel analysis identified additional subpopulations among *F. tularensis* subsp. *holarctica* strains. Geographic origins of these subpopulations suggest dispersal over large distances. Two strains from the United States, OSU18 (represented by genome sequence data only) and FSC035, were identical at all indel loci and constitute a distinct genetic entity. Strains FSC012 from the United States and FSC519 from Sweden formed another entity. Finally, 6 strains originating in Sweden or Russia represented a third subpopulation. Compared with indel analysis, MLVA showed much more extensive polymorphisms, which was helpful for characterizing individual strains. Simpson’s index of diversity ranged between 0.17 and 0.97 for the MLVA loci and between 0.09 and 0.52 for the indel loci, which reflects the fact that only 2 allele states were present for the indel loci while the MLVA loci were more diverse, with up to 16 alleles (for MLVA marker Ft-M3).

### Phylogenetic Inferences Based on MLVA and Indel Data

**Figure 4:**
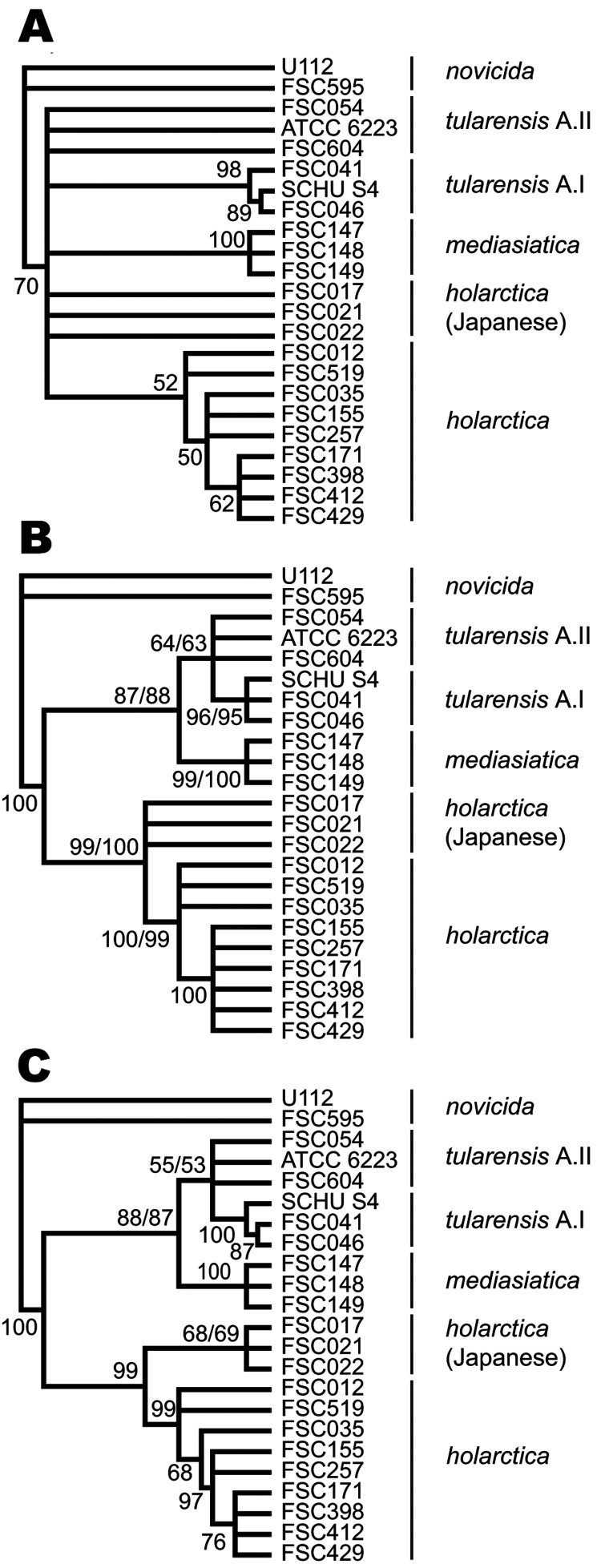
Cladograms depicting relationships among *Francisella tularensis* strains obtained by maximum parsimony and bootstrap analysis that used indel, multilocus variable-number tandem repeat analysis (MLVA), or combined data. Nodes supported by <50% of bootstrap pseudoreplicates were collapsed. A) Cladogram obtained solely from the use of MLVA data. B) Cladogram from the use of indel data. C) Cladogram from the combined use of indel and MLVA data. The dual bootstrap support values presented represent the use of each of 2 alleles, found at locus Ftind-32 of strain ATCC 6223.

Genetic relationships among *F. tularensis* strains were inferred by MP analysis of the MLVA data, indel data, or both indel and MLVA data (Figure 4). The use of MLVA data alone resulted in weak support for delineation of deeper branching patterns, few nodes having >50% support in bootstrap analysis (Figure 4, panel A). For such purposes, indel data alone were more valuable (Figure 4, panel B). The use of combined indel and MLVA data resulted in well-supported deep nodes and discrimination of the strains included in this study (Figure 4, panel C).

In strain ATCC 6223, dual bootstrap support values (Figure 4, panels B, C) represent values obtained by using each of the 2 alleles amplified for locus Ftind-32. The same topology was obtained regardless of which allele was included, and the allele used had minor effect on bootstrap support values. Results were highly similar when using inference by neighbor joining (data not shown).

## Discussion

By combining canonical indels with MLVA, robust subspecies and major clade typing of *F. tularensis* was successfully combined with high-resolution typing among strains. By the use of killed bacterial preparations, the 2 marker sets were rapidly assayed by fragment analysis.

The present canonical indel/MLVA typing concept adapts well to the principles of diagnostic work inherent in public health laboratories. The concept generates portable straight numeric data and, similar to the tests of biochemical reactions, 2 alternative states are determined at multiple indels (Figure 3). The MLVA output consists of multistate discrete numbers and has proven superior to PFGE for reliable resolving of discrete strains of the species (*6*,*7*).

Typing of *F. tularensis* provides useful public health information. This is especially relevant to North America, where subpopulations varying in virulence occur naturally in the same geographic region. According to a recent report, major genetic subpopulations within the type A tularemia population (A.I and A.II) seem connected with different mortality rates in humans (*7*). Potential clinical correlates to type B subpopulations remain to be studied. Ongoing work shows that >90 European isolates all fall within the subpopulations described here (unpub. data).

A most conspicuous need for rapid and reliable characterization of isolates of *F. tularensis* relates to bioterrorism. Whenever tularemia appears in an area believed to be free from the agent, characterization of isolates will become urgent. Such characterization abilities may also prove useful in understanding how *F. tularensis* may spread under peaceful circumstances. Reminders of the agent’s potential for infection include the unexplained introduction of the disease on Martha’s Vineyard in 1937 and more recently in northern Spain in 1997–1998, along with the highly publicized 2004 laboratory infections with respiratory type A tularemia at Boston University (*5*,*17*,*34*).

In public health laboratories, indel/MLVA typing may replace more risky and time-consuming biochemical characterization, which is based on growth of *F. tularensis*. After initial culture of the agent, noninfectious DNA is rapidly analyzed by PCR and fragment analysis for determination of indel and MLVA data.

A major achievement of the present study was the identification of canonical indels for combined use with MLVA. From studies of *Bacillus* spp., only SNPs have been predicted to exhibit mutation rates sufficiently slow to be useful for unambiguous assignment of bacteria at deeper taxonomic levels (*24*). SNPs with canonical properties are not yet recognized in *F. tularensis,* and their combined use with MLVA has thus not yet been evaluated. An SNP-based approach does conform with well-developed evolutionary models to support data analysis (*35*), models that do not exist for indel mutations. A drawback is, however, that the involvement of 2 different analytic methods in a combined MLVA/SNP-based analysis makes it more complicated. By use of fragment analysis for both steps, the indel/MLVA approach is more effective. This study indicates that canonical indels can be integrated into evolutionary analyses for measuring large genetic distances while MLVA provides a detailed examination at short distances.

When selecting indels for the presented typing procedure, we took precautions to avoid DNA-marker discovery bias and homoplastic markers, problems that had been carefully addressed in work on other bacterial pathogens (*36*,*37*). To minimize discovery bias, we used *F. tularensis* genomes classified as being distantly related by independent methods. Genomes selected represented all 4 subspecies of *F. tularensis* that also form major genetic clades, according to MLVA, PFGE, microarray, and various arbitrarily primed–PCR analyses (*1*). To avoid homoplasy, including gene conversion, we excluded indels associated with repeat sequences. Our genome sequence data and the overall tree structure obtained from analysis of indel data lent support to a paucity of homoplasy effects. Except for locus Ftind-32 in the type strain ATCC 6223, which exhibited 2 PCR amplicons, only 4 of 280 identified loci showed incongruent evolutionary allele patterns. These 4 loci were all found among those repeat-containing loci that were excluded according to our selection criteria.

The reason behind a deviant result of strain ATCC 6223 at 1 locus is unknown but may be related to laboratory-induced mutations. ATCC 6223 was originally isolated in 1920 from a human lymph node in Utah, became avirulent by laboratory passage in the early years, but still retained properties that made it useful for antigen production. Recent microarray studies showed that it lacks portions of the genetic repertoire shared by all other *F. tularensis* strains (*10*).

MLVA discriminates among individual isolates within subspecies but may cause false estimates of relationships at deeper phylogenetic levels. Although in a previous study that used the present 25-marker MLVA scheme, discrimination of *F. tularensis* subspecies and major genetic clades was achieved, bootstrap support at these deeper levels was weak (*6*). Also in the present study, deep structural relationships among strains inferred by MP analysis of MLVA data were found to be weakly resolved. Conversely, strong support was shown for deep-level nodes obtained by using indel data. A combined analysis with both MLVA and indel data retained the deep-level support and yielded the most resolved topology. Furthermore, despite the inability of the indel or MLVA data to provide support for a separate clade of Japanese strains, such separation was supported by the combined analysis. This demonstrates that topologic constraints imposed by canonical indel data reduced the number of alternative positions of a combined tree and consequently increased the support for a clade.

When the present approach is used for routine purposes, the number of DNA markers might well be reduced yet retain a high level of discrimination and robustness. However, such a reduction needs to be evaluated to ensure proper marker selection. The inclusion by international collaboration of large numbers of geographically distributed strains will be facilitated by the unambiguous nature of data collected and the use of low quantities of killed bacteria. For ordinary clinical purposes, only a few indel markers may be required to rapidly receive relevant information, i.e., whether an isolate belongs to a subspecies or major genetic clade. A reference laboratory may wish to add more markers for tracing outbreaks and for forensic applications. Tailored combinations of these markers can be easily integrated into multiplex assays with 4–8 markers per PCR amplification and subsequent multicolor fragment analysis to decrease analytical time and cost.

In essence, we used 5 genome sequences representative of the species *F. tularensis* to identify 158 canonical indel DNA-markers, of which 38 were selected to provide robust information specific to each major genetic clade. By combining analysis of these indel markers with MLVA, discrimination of individual strains was achieved. The usefulness of indels with canonical properties may not be restricted to *F. tularensis*. The current availability of multiple genome sequences should allow testing this typing strategy for other clinically relevant pathogens.

## Supplementary Material

Appendix Table 1 Francisella tularensis genomic sequences used for indel identification

Appendix Table 2 Francisella tularensis strains and PCR-amplicon sizes at 38 indel loci*

Appendix Table 3 Francisella tularensis strains and repeat copy number at 25 MLVA loci*
