# Supplementary material for: Canonical Insertion-Deletion Markers for Rapid DNA Typing of Francisella tularensis
Source: Emerg Infect Dis. 2007 Nov;13(11):1725–32. doi: 10.3201/eid1311.070603 (PMC2874433; doi:10.3201/eid1311.070603)
Supplement: Appendix Table 1 — Francisella tularensis genomic sequences used for indel identification [file 07-0603_appT1-s1.pdf]

Appendix Table 1. *Francisella tularensis* genomic sequences used for indel identification

F. tularensis subspecies	Strain	FSC* no.	ATCC† no.	Source of genomic sequence	Status	GenBankaccession no.	Reference
novicida	U112	040‡	15482	University of Washington, Seattle, WA, USA	Finished	CP000439	Unpub.
tularensis	SCHU S4	237	–	Swedish/British/US consortium§	Finished	AJ749949	(*1*)
mediasiatica	GIEM 543	147	–	Los Alamos National Laboratory, Los Alamos, NM, USA	Unfinished	Pending	Unpub.
holarctica	OSU18	–	–	Baylor College of Medicine, Waco, TX, USA	Finished	CP000437	(*2*)
holarctica	LVS	458‡	29684	Lawrence Livermore National Laboratory, CA, USA	Finished	AM233362	Unpub.

**Francisella* Strain Collection.

†American Type Culture Collection.

‡Strains sequenced were not obtained from the *Francisella* Strain Collection.

§Member institutes of the sequencing consortium were: Swedish Defence Research Agency, Sweden; Uppsala and Umeå Universities, Sweden, Defence Science and Technology Laboratories, Salisbury, UK; Walter Reed Army Institute of Research, Bethesda, Maryland, USA.

References

1. Sandström G, Tärnvik A, Wolf-Watz H, Löfgren S. Antigen from *Francisella tularensis*: nonidentity between determinants participating in cell-mediated and humoral reactions. Infect Immun. 1984;45:101–6.Medline
2. Petrosino JF, Xiang Q, Karpathy SE, Jiang H, Yerrapragada S, Liu Y, et al. Chromosome rearrangement and diversification of *Francisella tularensis* revealed by the type B (OSU18) genome sequence. J Bacteriol. 2006;188:6977–85.
