# Supplementary material for: Canonical Insertion-Deletion Markers for Rapid DNA Typing of Francisella tularensis
Source: Emerg Infect Dis. 2007 Nov;13(11):1725–32. doi: 10.3201/eid1311.070603 (PMC2874433; doi:10.3201/eid1311.070603)
Supplement: Appendix Table 2 — Francisella tularensis strains and PCR-amplicon sizes at 38 indel loci* [file 07-0603_appT2-s2.pdf]

Appendix Table 2. *Francisella tularensis* strains and PCR-amplicon sizes at 38 indel loci*

Species (genetic clade), source	FSC no.	Other designation	Amplified DNA size (bp) at indel locus*									
			Ftind1	Ftind2	Ftind3	Ftind4	Ftind5	Ftind6	Ftind7	Ftind8	Ftind9	Ftind10
F. tularensis subsp. *novicida*												
Water, 1950, Utah	040	U112, ATCC 15482	316	288	387	228	202	394	382	380	388	286
Human, 2004, UK-Germany-Brazil	595	F58	292	355	388	230	205	394	382	380	389	285
F. tularensis subsp. *tularensis* (A.II)												
Rabbit, 1953, Nevada	054	Nevada 14	293	354	376	236	216	350	372	375	389	285
Human lymph node, 1920, Utah	230	ATCC6223	292	354	376	237	216	351	372	375	388	285
Foal, 1959, Montana	604	8859	293	355	377	237	216	350	372	375	388	284
F. tularensis subsp. *tularensis* (A.I)												
Human ulcer, 1941, Ohio	237	SCHU S4	292	354	376	236	215	351	373	374	270	281
Tick, 1935, British Columbia, Canada	041	Vavenby	293	354	377	236	216	350	372	374	269	281
Human pleural fluid, 1940, Ohio	046	Fox Downs	292	354	376	237	217	350	372	375	270	280
F. tularensis subsp. *mediasiatica*												
Miday gerbil, 1965, Kazakhstan	147	GIEM 543	292	354	376	237	216	346	372	374	388	285
Tick, 1982, Central Asia	148	240	292	354	376	236	216	346	372	374	388	286
Hare, 1965, Central Asia	149	120	291	354	376	237	216	346	372	374	389	285
F. tularensis subsp. *holarctica* (Japanese)												
Human lymph node, 1926, Japan	017	S-2	292	354	374	237	216	350	372	374	388	285
Human, 1958, Japan	021	Tsuchiya	293	354	374	236	216	350	372	374	388	286
Human, 1950, Japan	022	Ebina	292	354	374	236	216	351	372	374	388	285
F. tularensis subsp. *holarctica* (non-Japanese)												
Beaver, 1976, Hamilton, Montana	035	B423A	293	354	377	236	215	351	373	374	388	286
Tick, 1941, Montana	012	425F4G	293	354	375	237	216	351	372	374	388	286
Human, 2004, Örebro, Sweden	519		293	355	377	236	216	350	372	373	388	285
Live vaccine strain, Russia	458	LVS, ATCC 29684	292	354	376	236	215	351	373	374	388	285

Human, 1995, Ljusdal, Sweden	171		292	354	376	237	216	350	372	374	388	285
Human, 2003, Örebro, Sweden	398		292	354	376	237	216	350	372	373	388	285
Human, 2003, Örebro, Sweden	412		292	354	376	236	216	351	372	373	388	285
Human, 2003, Örebro, Sweden	429		293	355	377	236	216	350	372	373	388	285
Tick, 1949, Moscow area, Russia	257	GIEM 503/840	293	355	377	237	216	350	372	373	388	285

			Amplified DNA size (bp) at indel locus*									
Species (genetic clade), source	FSC no.	Other designation	Ftind11	Ftind12	Ftind13	Ftind14	Ftind15	Ftind16	Ftind17	Ftind18	Ftind19	Ftind20
F. tularensis subsp. *novicida*												
Water, 1950, Utah	040	U112, ATCC 15482	317	288	274	268	249	268	212	225	171	337
Human, 2004, UK-Germany-Brazil	595	F58	317	287	279	268	249	268	212	225	171	336
Francisella tularensis subsp. *tularensis* (A.II)												
Rabbit, 1953, Nevada	054	Nevada 14	317	273	264	256	249	268	212	225	171	336
Human lymph node, 1920, Utah	230	ATCC6223	317	273	263	256	249	268	212	225	171	336
Foal, 1959, Montana	604	8859	317	272	264	256	249	268	212	225	171	337
F. tularensis subsp. *tularensis* (A.I)												
Human ulcer, 1941, Ohio	237	SCHU S4	279	272	262	256	249	268	212	225	171	337
Tick, 1935, British Columbia, Canada	041	Vavenby	279	272	263	256	249	268	212	225	171	337
Human pleural fluid, 1940, Ohio	046	Fox Downs	279	272	262	256	249	268	212	225	171	337
F. tularensis subsp. *mediasiatica*												
Miday gerbil, 1965, Kazakhstan	147	GIEM 543	317	289	262	256	234	243	192	203	145	337
Tick, 1982, Central Asia	148	240	317	268	263	256	234	243	192	203	145	336
Hare, 1965, Central Asia	149	120	317	289	262	256	234	243	192	203	145	336
F. tularensis subsp. *holarctica* (Japanese)												
Human lymphnode, 1926, Japan	017	S-2	317	288	274	267	250	268	212	225	171	323
Human, 1958, Japan	021	Tsuchiya	318	288	275	267	250	268	212	225	171	323
Human, 1950, Japan	022	Ebina	317	288	274	267	250	268	212	225	171	323

F. tularensis subsp. *holarctica* (non-Japanese)												
Beaver, 1976, Hamilton, Montana	035	B423A	317	288	274	267	249	268	212	225	171	324
Tick, 1941, Montana	012	425F4G	318	288	274	267	249	268	212	225	171	323
Human, 2004, Örebro, Sweden	519		317	288	273	267	249	268	212	225	171	323
Live vaccine strain, Russia	458	LVS, ATCC 29684	317	289	273	268	249	268	212	225	171	324
Human, 1995, Ljusdal, Sweden	171		317	288	273	267	249	268	212	225	171	324
Human, 2003, Örebro, Sweden	398		317	287	273	267	249	268	212	225	171	324
Human, 2003, Örebro, Sweden	412		317	288	274	267	249	268	212	225	171	323
Human, 2003, Örebro, Sweden	429		317	288	273	267	249	268	212	225	171	323
Tick, 1949, Moscow area, Russia	257	GIEM 503/840	317	288	273	267	249	268	212	225	171	324

			Amplified DNA size (bp) at indel locus*									
Species (genetic clade), source	FSC no.	Other designation	Ftind21	Ftind22	Ftind23	Ftind24	Ftind25	Ftind26	Ftind27	Ftind28	Ftind29	Ftind30
F. tularensis subsp. *novicida*												
Water, 1950, Utah	040	U112, ATCC 15482	352	345	241	341	348	347	339	337	338	352
Human, 2004, UK-Germany-Brazil	595	F58	352	347	241	341	349	346	339	337	337	351
F. tularensis subsp. *tularensis*, (A.II)												
Rabbit, 1953, Nevada	054	Nevada 14	353	345	241	341	349	347	341	337	337	351
Human lymph node, 1920, Utah	230	ATCC6223	353	345	242	341	349	347	341	337	338	351
Foal, 1959, Montana	604	8859	353	346	241	341	349	346	341	337	338	351
F. tularensis subsp. *tularensis*, (A.I)												
Human ulcer, 1941, Ohio	237	SCHU S4	352	346	242	341	348	347	340	337	338	352
Tick, 1935, British Columbia, Canada	041	Vavenby	352	345	241	341	348	347	340	336	338	351
Human pleural fluid, 1940, Ohio	046	Fox Downs	353	345	242	341	349	348	341	337	338	352
F. tularensis subsp. *mediasiatica*												
Miday gerbil, 1965, Kazakhstan	147	GIEM 543	353	346	242	341	349	347	340	337	337	351
Tick, 1982, Central Asia	148	240	352	346	241	340	349	347	340	337	337	351

Hare, 1965, Central Asia	149	120	352	346	241	340	349	347	340	337	337	351
F. tularensis subsp. *holarctica* (Japanese)												
Human lymph node, 1926, Japan	017	S-2	342	312	228	336	349	347	340	337	338	351
Human, 1958, Japan	021	Tsuchiya	343	312	228	336	349	347	340	337	339	351
Human, 1950, Japan	022	Ebina	343	312	228	336	349	347	340	337	338	351
F. tularensis subsp. *holarctica* (non-Japanese)												
Beaver, 1976, Hamilton, Montana	035	B423A	342	312	230	336	345	284	315	310	331	352
Tick, 1941, Montana	012	425F4G	343	312	228	336	344	284	315	311	330	351
Human, 2004, Örebro, Sweden	519		343	312	228	336	343	284	315	310	330	351
Live vaccine strain, Russia	458	LVS, ATCC 29684	343	312	229	336	344	284	315	311	331	346
Human, 1995, Ljusdal, Sweden	171		343	313	228	336	344	284	315	310	331	346
Human, 2003, Örebro, Sweden	398		342	313	228	336	343	284	315	311	330	346
Human, 2003, Örebro, Sweden	412		343	312	228	336	344	284	315	310	330	346
Human, 2003, Örebro, Sweden	429		342	312	228	336	344	284	315	310	330	346
Tick, 1949, Moscow area, Russia	257	GIEM 503/840	342	312	228	336	343	284	315	311	330	346

			Amplified DNA size (bp) at indel locus*						
Species (genetic clade), source	FSC no.	Other designation	Ftind31	Ftind32	Ftind33	Ftind34	Ftind35	Ftind36	Ftind37
F. tularensis subsp. *novicida*									
Water, 1950, Utah	040	U112, ATCC 15482	328	334	315	308	388	244	398
Human, 2004, UK-Germany-Brazil	595	F58	327	335	315	308	386	244	397
F. tularensis subsp. *tularensis* (A.II)									
Rabbit, 1953, Nevada	054	Nevada 14	325	334	315	308	387	244	399
Human lymph node, 1920, Utah	230	ATCC6223	325	324 334	315	308	388	244	398
Foal, 1959, Montana	604	8859	325	335	314	308	387	244	398
F. tularensis subsp. *tularensis* (A.I)									
Human ulcer, 1941, Ohio	237	SCHU S4	325	335	314	305	388	244	399

Tick, 1935, British Columbia, Canada	041	Vavenby	325	334	315	308	387	244	397
Human pleural fluid, 1940, Ohio	046	Fox Downs	325	335	315	308	387	244	399
F. tularensis subsp. *mediasiatica*									
Miday gerbil, 1965, Kazakhstan	147	GIEM 543	324	334	315	308	388	243	398
Tick, 1982, Central Asia	148	240	325	334	315	308	387	244	399
Hare, 1965, Central Asia	149	120	325	334	315	308	387	244	398
F. tularensis subsp. *holarctica* (Japanese)									
Human lymph node, 1926, Japan	017	S-2	325	334	315	308	387	244	398
Human, 1958, Japan	021	Tsuchiya	325	334	315	308	388	245	399
Human, 1950, Japan	022	Ebina	325	334	315	308	387	244	398
F. tularensis subsp. *holarctica* (non-Japanese)									
Beaver, 1976, Hamilton, Montana	035	B423A	326	334	315	308	371	236	391
Tick, 1941, Montana	012	425F4G	325	334	315	308	388	244	398
Human, 2004, Örebro, Sweden	519		325	334	315	308	387	244	397
Live vaccine strain, Russia	458	LVS, ATCC 29684	321	324	296	268	388	244	398
Human, 1995, Ljusdal, Sweden	171		320	324	295	268	387	244	398
Human, 2003, Örebro, Sweden	398		319	325	295	268	387	244	399
Human, 2003, Örebro, Sweden	412		320	324	295	268	387	244	399
Human, 2003, Örebro, Sweden	429		320	324	294	266	387	244	397
Tick, 1949, Moscow area, Russia	257	GIEM 503/840	319	324	295	266	387	243	399

*Size determinations include a 19-bp M-13 primer tail sequence used for fluorescent labeling. Ftindel, *F. tularensis* insertion-deletion loci; FSC, *Francisella* Strain Collection; ATCC, American Type Culture Collection.
