# Supplementary material for: Canonical Insertion-Deletion Markers for Rapid DNA Typing of Francisella tularensis
Source: Emerg Infect Dis. 2007 Nov;13(11):1725–32. doi: 10.3201/eid1311.070603 (PMC2874433; doi:10.3201/eid1311.070603)
Supplement: Appendix Table 3 — Francisella tularensis strains and repeat copy number at 25 MLVA loci* [file 07-0603_appT3-s3.pdf]

Appendix Table 3. *Francisella tularensis* strains and repeat copy number at 25 MLVA loci*

Species (genetic clade), source	FSC no.	Other designation	Repeat copy no. at MLVA locus																								
			Ft-M1	Ft-M2	Ft-M3	Ft-M4	Ft-M5	Ft-M6	Ft-M7	Ft-M8	Ft-M9	Ft-M10	Ft-M11	Ft-M12	Ft-M13	Ft-M14	Ft-M15	Ft-M16	Ft-M17	Ft-M18	Ft-M19	Ft-M20	Ft-M21	Ft-M22	Ft-M23	Ft-M24	Ft-M25
F. tularensis subsp. *novicida*																											
Water, 1950, Utah	040	U112, ATCC 15482	3	2	32	5	2	4	2	2	4	2	4	2	2	3	3	1	2	2	1	31	2	4	1	1	5
Human, 2004, UK-Germany-Brazil	595	F58	3	2	20	5	2	4	2	1	2	2	4	2	2	4	3	1	2	2	1	31	2	4	1	1	5
F. tularensis subsp. *tularensis* (A.II)																											
Rabbit, 1953, Nevada	054	Nevada 14	3	21	16	3	2	4	2	2	2	1	5	2	1	3	3	1	2	2	1	22	2	3	1	1	5
Human lymph node, 1920, Utah	230	ATCC6223	3	25	8	3	2	4	2	4	2	1	5	2	1	3	3	1	2	2	1	25	2	3	1	1	5
Foal, 1959, Montana	604	8859	3	7	11	4	2	4	2	2	2	1	5	2	1	4	3	1	2	2	1	21	3	3	1	1	5
F. tularensis subsp. *tularensis*, (A.I)																											
Human ulcer, 1941, Ohio	237	SCHU S4	3	4	25	3	3	4	4	4	4	20	5	2	2	3	2	2	3	4	1	3	3	2	2	1	5
Tick, 1935, British Columbia, Canada	041	Vavenby	3	24	19	3	2	4	3	2	5	10	5	2	2	3	2	2	3	4	1	3	2	2	1	1	5
Human pleural fluid, 1940, Ohio	046	Fox Downs	3	4	27	3	5	5	3	3	5	9	5	2	2	3	2	2	3	4	1	3	3	2	2	1	5
F. tularensis subsp. *mediasiatica*																											
Miday gerbil, 1965, Kazakhstan	147	GIEM 543	4	2	28	3	6	2	2	1	3	2	3	1	1	2	3	1	2	2	1	7	2	7	1	1	5
Tick, 1982, Central Asia	148	240	4	2	25	3	10	3	2	1	3	2	3	1	1	2	3	1	2	2	1	6	2	14	1	1	5
Hare, 1965, Central Asia	149	120	4	2	27	3	6	2	2	1	3	2	3	1	1	2	3	1	2	2	1	7	2	7	1	1	5
F. tularensis subsp. *holarctica* (Japanese)																											
Human lymph node, 1926, Japan	017	S-2	3	24	3	5	2	4	2	3	2	4	5	2	1	3	2	1	2	2	0	18	3	3	1	1	5
Human, 1958, Japan	021	Tsuchiya	3	8	3	5	2	5	2	1	2	8	5	2	1	3	3	1	2	2	0	11	2	3	1	1	5

Human, 1950, Japan	022	Ebina	3	12	3	5	2	4	1	4	2	4	5	2	1	3	3	1	2	2	0	11	2	3	1	1	5
F. tularensis subsp. *holarctica* (non-Japanese)																											
Beaver, 1976, Hamilton, Montana	035	B423A	3	2	19	6	2	5	2	2	2	2	5	2	1	3	3	1	2	2	0	3	2	3	1	2	4
Tick, 1941, Montana	012	425F4G	3	2	18	5	2	7	2	2	2	2	5	2	1	3	3	1	2	2	0	3	2	3	1	1	4
Human, 2004, Örebro, Sweden	519		3	2	12	5	4	4	2	2	2	2	5	2	1	4	3	1	2	2	0	3	2	3	1	1	4
Live vaccine strain, Russia	458	LVS, ATCC 29684	3	2	16	5	2	4	2	2	2	2	5	2	1	3	3	1	2	2	0	4	2	4	1	2	4
Human, 1995, Ljusdal, Sweden	171		3	2	10	5	2	5	2	2	2	2	5	2	1	4	3	1	2	2	0	3	2	4	1	2	4
Human, 2003, Örebro, Sweden	398		3	2	21	5	2	5	2	2	2	2	5	2	1	4	3	1	2	2	0	3	2	4	1	2	4
Human, 2003, Örebro, Sweden	412		3	2	21	5	2	5	2	2	2	2	5	2	1	4	3	1	2	2	0	3	2	4	1	2	4
Human, 2003, Örebro, Sweden	429		3	2	22	5	2	5	2	2	2	2	5	2	1	4	3	1	2	2	0	3	2	4	1	2	4
Tick, 1949, Moscow area, Russia	257	GIEM 503/840	3	2	17	5	2	4	2	2	2	2	5	2	1	3	3	1	2	2	0	3	4	4	1	2	4

*MLVA, multilocus variable-number tandem repeat analysis; FSC, *Francisella* Strain Collection; ATCC, American Type Culture Collection.
